# *Coxiella burnetii* and *Bartonella* Endocarditis Diagnosed by Metagenomic Next-Generation Sequencing

**DOI:** 10.3390/jcm11237150

**Published:** 2022-12-01

**Authors:** Weiteng Wang, Oudi Chen, Weijiang Liu, Lixi Gan, Xin Li, Qingyan Ma, Xuejiao Hu, Xuhua Jian

**Affiliations:** 1 Department of Cardiovascular Surgery, Guangdong Cardiovascular Institute, Guangdong Provincial People’s Hospital, Guangdong Academy of Medical Sciences, Guangzhou 510060, China; 2 Department of Echocardiography, Guangdong Cardiovascular Institute, Guangdong Provincial People’s Hospital, Guangdong Academy of Medical Sciences, Guangzhou 510060, China; 3 Department of Laboratory Medicine, Guangdong Provincial People’s Hospital, Guangdong Academy of Medical Sciences, Guangzhou 510060, China

**Keywords:** culture-negative endocarditis, *Coxiella burnetii*, *Bartonella*, metagenomic next-generation sequencing

## Abstract

(1) Background: Culture-negative endocarditis is challenging to diagnose. Here, we retrospectively identified 23 cases of *Coxiella burnetii* and *Bartonella* endocarditis by metagenomic next-generation sequencing. (2) Methods: Twenty-three patients with culture-negative endocarditis were retrospectively enrolled from Guangdong Provincial People’s Hospital (*n* = 23) between April 2019 and December 2021. Metagenomic next-generation sequencing was performed on blood (*n* = 22) and excised cardiac valvular tissue samples (*n* = 22) for etiological identification, and Sanger sequencing was performed for pathogenic diagnostic verification. The demographic and clinical data of the 23 patients were obtained from hospital electronic health records. (3) Results: A total of 23 male patients (median age, 56 years (interquartile range, 16)) with culture-negative endocarditis were diagnosed with *Coxiella burnetii* (*n* = 21) or *Bartonella* (*n* = 2) species infection by metagenomic next-generation sequencing. All patients underwent cardiac surgery. The resected tissue exhibited both a significantly higher number of unique suspected pathogen read-pairs and more unique pathogen read-pairs than the blood specimens. The results of Sanger sequencing tests on all remaining tissue and blood specimens were positive. Oral doxycycline was added to the antibiotic regimen for at least 1.5 years according to etiology. A total of 21 patients (91%) were discharged, and 20 patients were healthy at the 21-month (interquartile range, 15) follow-up visit. One patient exhibited endocarditis relapse with the same pathogen from inadequate antibiotic administration. The last 2 patients (9%) developed septic shock and multiple organ dysfunction syndrome postoperatively and died shortly after discharge. (4) Conclusions: CNE caused by *C. burnetii* and *Bartonella* species is challenging to diagnose and exhibits poor outcome due to delayed treatment. In response, mNGS, characterized by high sensitivity and rapid results, is an effective alternative for the etiological identification of *C. burnetii* and *Bartonella* endocarditis.

## 1. Introduction

Culture-negative endocarditis (CNE) is becoming more common in endocarditis patients, especially in tertiary hospitals. CNE has relatively high incidence rates of 15–40% in all cases of infective endocarditis and is an independent predictor of in-hospital mortality [1,2]. *Coxiella burnetii* is the most common causative pathogen of CNE (46.0–61.4%), followed by *Bartonella* species (15.1–20.7%) [3,4,5]. *C. burnetii* is a fastidious, Gram-negative and obligate intracellular bacterium, while *Bartonella* species are fastidious, Gram-negative, facultative intracellular bacteria with a unique intraerythrocytic lifestyle. This lifestyle not only enables the escape of their detection by the host immune system but can also cause false negative results on conventional laboratory tests [5]. The recommended major criteria of the modified Duke criteria for the diagnosis of *C. burnetii* endocarditis rely on an immunofluorescence assay (IFA) titer of ≥1:800 for *C. burnetii* anti-phase I immunoglobulin G (IgG). Serological cross-reactions between *C. burnetii*, *Bartonella* and *Chlamydia* [6,7] and inconvenient sample transfers to reference laboratories limit its clinical application. Due to the limitations of conventional laboratory tests and the unidentifiable clinical manifestations of these conditions, the delayed diagnosis or misdiagnosis of *C. burnetii* and *Bartonella* endocarditis are common and frequently result in inadequate treatment. To reach an etiological diagnosis and explore unique clinical manifestations in these particular patients, an alternative pathogenic assessment should be performed to achieve a correct and timely diagnosis. Here, we describe 23 cases of CNE caused by *C. burnetii* and *Bartonella* identified by metagenomic next-generation sequencing (mNGS).

## 2. Materials and Methods

## Ethics statement:

The study was approved by the ethics committee of the faculty of medicine at the Guangdong Provincial People’s Hospital (approve No. KY-Q-2022-173-01). Individual patients informed consents were all guaranteed except the death.

### 2.1. Patients

Between April 2019 and December 2021, twenty-three consecutive patients with C. burnetii or Bartonella endocarditis who underwent cardiac surgery at Guangdong Provincial People’s Hospital for valvular disease were included into this study. All patients met the modified Duke criteria for the clinical diagnosis of infective endocarditis [8]. The excised valvular tissue and blood specimens obtained during cardiac surgery were used to identify the pathogen by mNGS. Sanger sequencing was performed on the remaining specimens for etiologic verification. In addition, 31 patients with infective endocarditis caused by *Staphylococcus aureus* and 30 patients with infective endocarditis caused by *Streptococcus*, who were randomly selected from 258 infective endocarditis patients treated in our hospital between April 2019 and December 2021, were retrospectively studied as disease controls. All patient underwent cardiac surgery, and blood and tissue samples were subjected to mNGS for etiological identification.

### 2.2. Data Collection

The demographic and clinical data of the 84 patients were obtained from the electronic health records.

### 2.3. Metagenomic Next-Generation Sequencing

Plasma was prepared from the blood specimens, and circulating cell-free DNA (cfDNA) was isolated from the plasma with a QIAamp Circulating Nucleic Acid Kit (Qiagen, Hilden, Germany) according to the manufacturer’s protocol. DNA from the valvular tissue was extracted using the QIAamp DNeasy Blood & Tissue Kit (Qiagen, Hilden, Germany). The quantity and quality of DNA were assessed using a Qubit fluorometer (Thermo Fisher Scientific, Waltham, MA, USA) and a NanoDrop spectrophotometer (Thermo Fisher Scientific, Waltham, MA, USA), respectively. DNA libraries were prepared using the KAPA Hyper Prep kit (KAPA Biosystems, Oslo, Norway) according to the manufacturer’s protocols. An Agilent 2100 bioanalyzer was used for quality control, and DNA libraries were single-end sequenced (75 bp) on an Illumina NextSeq 550Dx system (Illumina, San Diego, CA, USA).

An in-house bioinformatics pipeline was used for pathogen identification. Briefly, high-quality sequencing data were generated by removing the low-quality reads, adapter contamination and duplicated and short (length < 36 bp) reads. Human host sequences were identified by mapping to the human reference genome (hs37d5) using Bowtie2 software. Reads that could not be mapped to the human genome were retained and aligned with the microbial genome database for pathogen identification. Our microbial genome database contained bacterial, fungal, viral and parasite genomic sequences (downloaded from https://www.ncbi.nlm.nih.gov/genome/) accessed on 1 February 2022.

The following criteria were used to define positive mNGS results:

For *Mycobacterium*, *Nocardia* and *Legionella pneumophila*, the result was considered positive if the data of a species detected by mNGS exhibited a species-specific read number ≥1.

For bacteria (excluding *Mycobacterium*, *Nocardia* and *Legionella pneumophila*), fungi, viruses and parasites, the result was considered positive if the data of a species detected by mNGS exhibited at least three nonoverlapping reads.

If the number of detected pathogens reads was ≥10-fold greater than that in the no template control (NTC), the pathogen detection was considered positive.

### 2.4. Organism-Specific Sanger Sequencing

Organism-specific Sanger sequencing was used for the verification of the mNGS results of the remaining specimens.

### 2.5. Statistical Analysis

All statistical analyses were performed with SPSS software, version 25.0 (IBM, Armonk, NY, USA). Continuous variables are summarized as the median with interquartile range (IQR). Categorical variables are summarized as frequencies and percentages. Comparisons were made between patients with infections caused by different pathogens. One-way ANOVA or the Welch test was used to compare continuous variables, and the chi-square test or Fisher’s exact test was used for categorical variables.

## 3. Results

All 23 patients with infections caused by *C. burnetii* or *Bartonella* were male, and the median age was 56 years (IQR, 16). The number of patients with blood cultures that were positive was significantly different among the *C. burnetii* and *Bartonella*, *Staphylococcus aureus* and *Streptococcus* groups (0, 0/23 vs. 23%, 7/31 vs. 20%, 6/30, *p* = 0.034). Four patients exhibited a fever higher than 38 °C, which was less than that caused by *Staphylococcus aureus* and *Streptococcus* (17%, 4/23 vs. 71%, 22/31 vs. 63%, 19/30, *p* < 0.001). Two patients (9%, 2/23) were previously diagnosed with CNE and received 4 weeks of intravenous antibiotic treatment that failed. Acute renal impairment was observed in 18 patients (78%, 18/23), including 7 patients (30%, 7/23) with severe renal injury and 11 (48%, 11/23) with moderate renal injury. Four patients (17%, 4/23) were admitted to the intensive care unit for poor cardiac function, sepsis shock and multiple organ dysfunction syndrome. Stable hemodynamics and mild infectious syndrome manifestations were observed in the remaining patients. Table 1 shows the demographic characteristics and outcome data of the three pathogenic groups.

Preoperative transthoracic echocardiography (TTE) and/or transesophageal echocardiography (TEE) demonstrated that the *C. burnetii* and *Bartonella* endocarditis groups exhibited more aortic valve vegetation than the two groups with endocarditis caused by *Staphylococcus aureus* and *Streptococcus* (96%, 22/23 vs. 61%, 19/31 vs. 47%, 14/30; *p* < 0.017), including 15 patients (65%, 15/23) who exhibited congenital bicuspid aortic valves and a less affected mitral valve (17%, 4/23 vs. 52%, 16/31 vs. 83%, 25/30; *p* < 0.017). The median diameter of the detected vegetation was 12.5 mm (IQR, 5.9), which was not significantly different among the three groups. Aortic regurgitation was present in 22 patients, including 19 patients (86%, 19/23) with severe regurgitation and 2 (9%, 2/23) with moderate regurgitation. Mitral valve regurgitation was diagnosed in 21 patients, including 9 patients (43%, 9/23) with severe regurgitation and 4 (19%, 4/23) with moderate regurgitation. Paravalvular abscess and fistula were more common in the *C. burnetii* or *Bartonella* species groups and the *Staphylococcus aureus* group than in the *Streptococcus* group (65%, 15/23 vs. 61%, 19/31 vs. 10%, 3/30, *p* < 0.001; 44%, 10/23 vs. 16%, 5/31 vs. 3%, 1/30, *p* < 0.001). Valve perforation was detected in 14 patients (61%, 14/23). The median left ventricular ejection fraction was 61% (IQR, 16%), which was not significantly different from those of the other pathogenic groups. Table 2 shows the echocardiographic and preoperative laboratory data of the three pathogen groups.

All 23 patients underwent cardiac surgery for progressively damaged cardiac valves. During the surgery, vegetation was observed in all patients and was completely resected. A total of 3 patients (13%) had aortic root aneurysms. Aortic valve replacement was performed in 22 patients (96%), and 6 patients (26%) underwent mitral valve replacement. A total of 8 patients (35%) underwent mitral valvuloplasty. A total of 6 patients (26%) underwent tricuspid valvuloplasty, and 1 underwent tricuspid bioprosthetic valve replacement (4%). Intra-aortic balloon pump surgery was performed in 3 patients (13%) because of poor left ventricular function. Just 1 patient (4%) underwent exploratory thoracotomy 9 h after the first cardiac surgery to drain excessive fluid in the mediastinum. All excised valve specimens were sent for tissue culture, but all tested negative, which was not significantly different among the other pathogenic groups (0, 0/23 vs. 13%, 4/31 vs. 7%, 2/30, *p* = 0.228).

A total of 21 patients (91%) were safely discharged, and targeted antibiotic therapy with doxycycline was continued for at least one and a half years. None required cardiac reoperation. Of those, 20 patients were healthy at the median 21-month (IQR, 15) follow-up visit. The most recent TTE scans performed on these 20 patients revealed no worsening of valvular leakage or valvular regurgitation. The remaining patient had a relapse of endocarditis in the 21st follow-up month. He only continued to adhere to doxycycline treatment for the first 6 months. Repeat blood culture in this patient was negative, but mNGS of blood revealed *C. burnetii* infection. The final 2 patients (9%) developed septic shock and multiple organ dysfunction syndrome postoperatively and died shortly after discharge.

**Figure 1:**
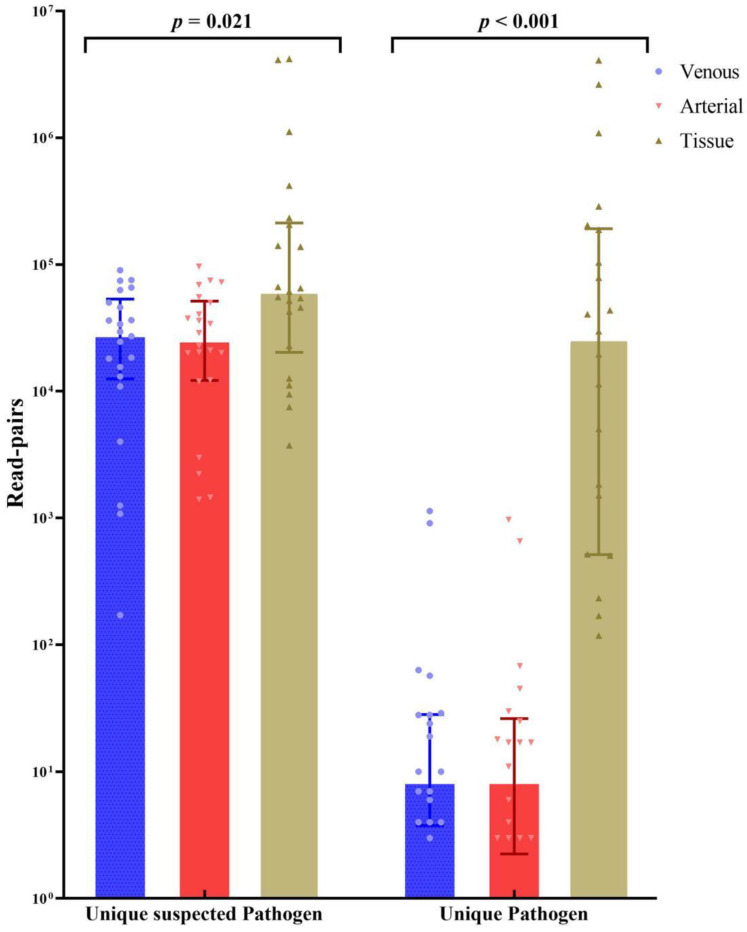
Unique suspected pathogen read-pairs and unique pathogen read-pairs sequenced in the venous blood specimens, arterial blood specimens and resected tissue. A unique suspected pathogen is defined as a unique nonhuman nonredundant sequence aligned with the microbial genome database. (The microbial genome can be downloaded from (The microbial genome of *Coxiella burnetii* and *Bartonella* can be downloaded from https://www.ncbi.nlm.nih.gov/sra/PRJNA853129 accessed on 30 June 2022) When the read-pairs of the unique suspected pathogen meet the positive mNGS criteria and the identity of the suspected pathogen is in accordance with clinical manifestations, the unique pathogen is defined. The resected tissue exhibited significantly higher numbers of both unique suspected pathogen read-pairs and unique pathogen read-pairs than the blood specimens by Kruskal–Wallis one-way ANOVA. (*p* = 0.021, *p* < 0.001).

**Figure 2:**
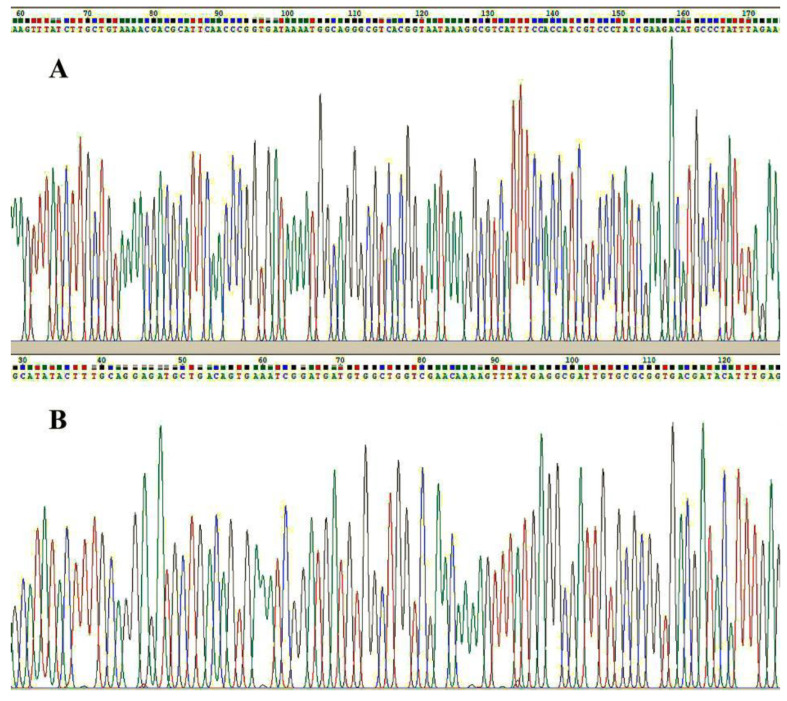
A unique DNA fragment of *Coxiella burnetii* was verified by Sanger sequencing in the resected tissue sample of Patient No. 5 (**A**). A unique DNA fragment of *Coxiella burnetii* was verified by Sanger sequencing in the resected tissue sample of Patient No. 5 (**A**). The PCR primers of *Coxiella burnetii* for verification were *TAACGCAAGGCGGTGATTTAG* and *CGATAGGGACGATGGTGGAA*. The sequence ID of the target species was CP040059.1, and the location of the target sequence was 1: 214,444 to 214,577. A unique DNA fragment of *Bartonella* species was verified by Sanger sequencing in the resected tissue sample of Patient No. 4 (**B**). The PCR primers of *Bartonella* species for verification were *TGGTGGTCAGCGTTTTGGT* and *CCTCAAATGTATCGTCACCGC*. The sequence ID of the target species was AP019773.1, and the location of the target sequence was 1: 687,822 to 687,945. The colored curves represented different basic group such as green for adenine, red for thymine, black for guanine and blue for cytosine.

The mNGS procedure was performed on the excised valve tissue of 22 patients (all except Patient No. 2), and the results showed *Bartonella* species infection in 2 patients (9%) and *C. burnetii* infection in 20 patients (91%), including a mixed infection with *Streptococcus gordonii* in 1 patient. The mNGS was performed on blood samples from 22 patients (all except Patient No. 4) and showed *C. burnetii* infection in 18 patients (82%, 18/22), *Bartonella* species infection in 2 patients (9%, 2/22) and additional *Streptococcus gordonii* infection in 1 patient (5%, 1/22). Patient No. 1, Patient No. 3 and Patient No. 13 exhibited positive mNGS results in the excised valve tissue but negative results in blood specimens. Patient No. 12 exhibited positive mNGS results in blood specimens but negative results in the excised valve tissue. The excised valve tissue exhibited higher levels of unique suspected pathogenic richness and unique pathogenic richness than the blood specimens (Figure 1). Sanger sequencing for *C. burnetii* or *Bartonella* species was performed on the remaining specimens to verify the mNGS results (Figure 2). All tissue and blood specimens tested for Sanger sequencing tested positive.

The case series of the *C. burnetii* and *Bartonella* species endocarditis groups are described in detail in Supplementary Materials S1.

## 4. Discussion

**Figure 3:**
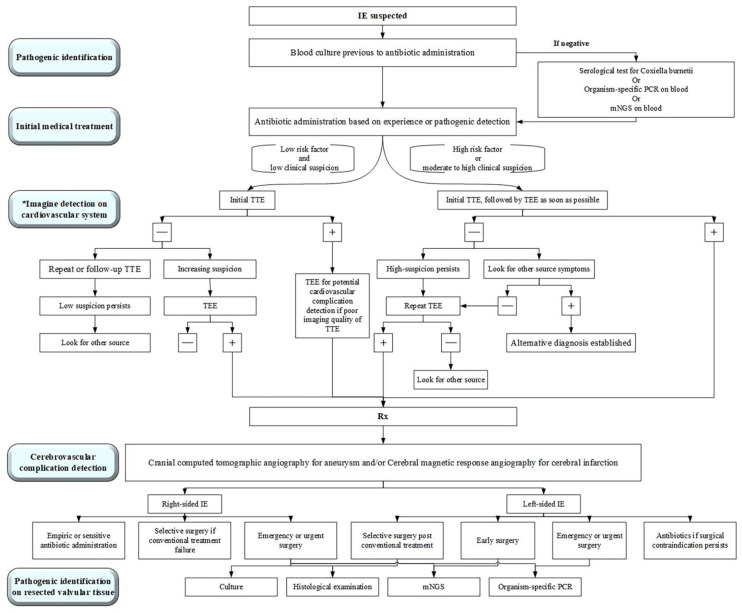
A simplified approach to the diagnosis and management of an infective endocarditis (IE) patient. Rx indicates prescription; TEE, transesophageal echocardiography; TTE, transthoracic echocardiography; mNGS, metagenomic next-generation sequencing; PCR, polymerase chain reaction; * Modified from Baddour et al. [10].

We presented 84 diagnostically challenging cases of acute or subacute endocarditis in which the use of mNGS on blood and valve tissue samples identified the pathogens involved. These cases included one case caused by *Bartonella quintana,* one case caused by *Bartonella henselae* and 21 cases caused by *C. burnetii,* including one patient who was also infected with *Streptococcus gordonii*. Sanger sequencing of the blood and valvular tissues was performed in these 23 patients for etiological verification. In Figure 3, we summarize the diagnosis and treatment process of endocarditis in our institute based on a 10-year experience of surgical management in infective endocarditis. Timely diagnosis, multiple disciplinary teams and effective antibiotic treatment [9] combined with surgery were key points for treatment.

Mild clinical manifestations of infection and severe levels of bacterial invasion and damage in the cardiac valve and aorta were characterized in patients with *C. burnetii* and *Bartonella* encarditis; in contrast, endocarditis caused by *Staphylococcus aureus* or *Streptococcus* was accompanied by evident sepsis that was easily recognized early. The severity and mortality of a patient’s condition can be predicted by clinicians using a variety of patient factors, including demographic, clinical, immunologic, hematologic, biochemical and radiographic findings, especially various laboratory test abnormalities and biomarkers of end-organ dysfunction [11]. Nonspecific symptoms of infection and a lack of pathogenic identification in *C. burnetii* and *Bartonella* endocarditis may decrease the vigilance of clinical physicians or surgeons, leading to delayed diagnosis or misdiagnosis and poor outcome [1,2]. Patients with *C. burnetiid* or *Bartonella* endocarditis may not undergo a cardiac examination or surgery until a new valvular regurgitation develops or the worsening of a preexisting valvular regurgitation occurs, which could indicate valve perforation, valve damage and/or, abscess [12]. Therefore, a rapid and effective diagnostic strategy is needed to reach the correct diagnosis of *C. burnetii* and *Bartonella* endocarditis and improve their outcome.

Unlike serological IFAs, molecular methods using the PCR-based amplification of pathogenic DNA fragments obtained from both blood and excised valve tissue prevent cross-reactions between biologically similar antigens. Of note, diagnoses of mixed pathogenic infections cannot be excluded in patients with *C. burnetii* or *Bartonella* endocarditis. Thus, the use of mNGS for broad pathogenic detection has diagnostic value compared with organism-specific PCR. However, mNGS exhibited lower specificity than that observed in conventional pathogenic identification examinations, including culture and serological IFAs, which may have resulted from contamination during the sample collection or molecular procedures or from the presence of chronic, persistent bacterial DNA in vivo [13]. Thus, mNGS results should be cautiously interpreted within the full clinical context and correlated with the findings of all other laboratory tests. The statistical scoring and filtering of microbiological and complex mNGS data sets can help inherently differentiate between microbiological contaminants and true infectious organisms. Sanger sequencing for pathogenic diagnostic verification was essential when the mNGS result was confusing. mNGS allows pathogenic identification within one working day, which facilitates timely diagnosis and the appropriate administration of antibiotics for CNE caused by *C. burnetii* and *Bartonella* species, which can improve the prognosis in a substantial number of patients.

**Figure 4:**
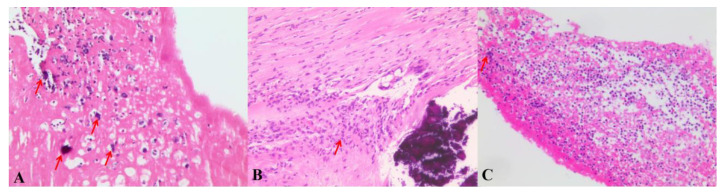
(**A**) Hematoxylin–eosin (H&E)-stained histological section of the mobile vegetation and congenital bicuspid aortic valve (Patient No. 3) showing fibrin-rich thrombi, infiltration of lymphocytes and macrophages and calcium deposition in *C. burnetii* endocarditis. Fewer neutrophiles were observed under the microscope. (**B**,**C**) Hematoxylin–eosin (H & E)-stained histological section of the mobile vegetation mixed with *Staphylococcus aureus* (**B**) and *Streptococcus* (**C**) showing a mass of neutrophiles infiltration and microorganisms (arrow).

Interestingly, we summarized the visual characteristics of *C. burnetii* and *Bartonella* endocarditis (Figure 4). Of note, the endomembrane of the abscess was smooth and integrated, and less exudate was detected in the abscess than in other paravalvular abscesses caused by *Staphylococcus aureus* or *Streptococcus*. This phenomenon may be related to the diversity of infection and the invasiveness of different bacterial strains [14]. Here, we offer three short surgical videos showing the differences in vegetation and paravalvular abscesses in different etiological forms of endocarditis (Supplementary Video S1). However, our study was an observational study with a small sample and was quite subjective for use in attending decisions. Additionally, the study only included endocarditis patients who underwent cardiac surgery for valve tissue acquisition. This is the natural bias in patient selection. Further study involving more specimens and a more rigorous design is required for the verification of our results.

## 5. Conclusions

CNE caused by *C. burnetii* and *Bartonella* species is challenging to diagnose and has a poor outcome due to delayed treatment. In response, mNGS, characterized by high sensitivity and rapid results, is an effective alternative method for the etiological identification of *C. burnetii* and *Bartonella* endocarditis.

## Figures and Tables

**Table 1:** Demographic and clinical characteristics of 84 patients.

	Total (*n* = 84)	*C. burnetii* and *Bartonella* (*n* = 23, 27.4%)	*Staphylococcus aureus* (*n* = 31, 36.9%)	*Streptococcus* (*n* = 30, 35.7%)	*p* Value
Male sex, *n* (%)	67 (80)	23 (100)	22 (71) #	22 (73) #	0.018
Age, y, median (IQR)	55 (26)	56 (16)	52 (28) *	55 (31)	0.029 &
Fever, *n* (%)	45 (54)	4 (17)	22 (71) #	19 (63) #	<0.001
LOS in hospital, d, median (IQR)	37 (27)	26 (28)	45 (20) *	30 (32)	0.003
Cost, RMB, thousand, *n* (IQR)	195 (115)	199 (91)	224 (184)	176 (86)	0.053
Classification of NYAH, *n* (%)					0.113
I-II	19 (23)	3 (13)	7 (23)	9 (30)	
III	39 (46)	14 (61)	10 (32)	15 (30)	
IV	26 (31)	6 (26)	14 (45)	6 (20)	
Native valve IE, *n* (%)	78 (93)	20 (87)	28 (90)	30 (100)	0.143
Prosthetic valve IE, *n* (%)	6 (7)	3 (13)	3 (10)	0	
Coronary artery disease, *n* (%)	24 (29)	10 (44)	6 (19)	8 (27)	0.146
Acute heart failure, *n* (%)	42 (50)	9 (39)	21 (68)	12 (40)	0.045
Arrythmia, *n* (%)	18 (21)	6 (26)	6 (19)	6 (20)	0.814
Renal impairment+					0.012
Moderate, *n* (%)	26 (31)	11 (48)	3 (10)	12 (40)	
Severe, *n* (%)	29 (35)	7 (30)	15 (48)	7 (23)	
Neurological complication, *n* (%)	29 (35)	5 (22)	13 (42)	11 (37)	0.290
Intracranial hemorrhage, *n* (%)	12 (14)	1 (4.3)	7 (23)	1 (13)	0.156
Embolism, *n* (%)	29 (35)	5 (22)	13 (42)	12 (40)	0.257
Cardiac surgery					
CPB, min, median (IQR)	160 (89)	170 (76)	163 (134)	155 (61)	0.049 &
ACC, min, median (IQR)	108 (58)	126 (48)	106 (74)	99 (43) *	0.023
IABP, *n* (%)	6 (7)	3 (13)	2 (6.5)	1 (3.3)	0.494
ECMO, *n* (%)	1 (1.2)	0	1 (3.2)	0	1
Short-term mortality, *n* (%)	6 (7.1)	2 (8.7)	1 (3.2)	3 (10)	0.573
Follow-up, months, median (IQR)	23 (15)	21 (14)	23 (12)	20 (19)	0.353
Follow-up survival, *n* (%)	78 (100)	21 (100)	30 (100)	27 (100)	
IE relapse, *n* (%)	2 (2)	1 (4)	1 (3)	0	0.733

Continuous data are presented as the median (IQR), and categorical data are presented as *n* (%). LOS Length of stay, NYHA New York Heart Association, IE infective endocarditis, CPB cardiac pulmonary bypass, ACC Aorta cross-clamp, IABP intra-aortic balloon pump, ECMO extracorporeal membrane oxygenation. +Renal impairment: there are now 3 categories based on levels of creatinine clearance calculated using the Cockcroft–Gault formula. The 3 categories were as follows: on dialysis (regardless of serum creatinine level); moderately impaired renal function (50–85 mL/min); severely impaired renal function (<50 mL/min); and off dialysis. * Compared with the *C. burnetii* and *Bartonella* endocarditis groups by Dunnett’s test, *p* < 0.05. # Compared with the *C. burnetii* and *Bartonella* endocarditis groups by the chi-square test, *p* < 0.017. & Welch test Additional demographic and clinical characteristics of these 23 cases are described in detail in Supplementary Materials S1.

**Table 2:** Echocardiographic and laboratory findings in 84 patients with infective endocarditis.

	Total (*n* = 84)	*C. burnetii* and *Bartonella* (*n* = 23, 27.4%)	*Staphylococcus aureus* (*n* = 31, 36.9%)	*Streptococcus* (*n* = 30, 35.7%)	*p* Value
Affected valve					
Left-side endocarditis	82 (98)	23 (100)	29 (93)	30 (100)	<0.001
Only aortic valve, *n* (%)	37 (44)	19 (83)	13 (42) *	5 (17) *	
Only mitral valve, *n* (%)	27 (32)	1 (4)	10 (32) *	16 (53) *	
Aortic valve and mitral valve, *n* (%)	18 (21)	3 (13)	6 (19)	9 (30)	
Right-side endocarditis	8 (10)	1 (4)	4 (13)	3 (10)	0.595
Only tricuspid valve, *n* (%)	8 (10)	1 (4)	2 (7)	3 (10)	
tricuspid valve and pulmonary valve, *n* (%)	2 (2)	0	2 (7)	0	
CABV, *n* (%)	30 (36)	15 (65)	11 (36)	4 (13) #	<0.001
Cardiac complication					
Abscess, *n* (%)	37 (44)	15 (65)	19 (61)	3 (10) #	<0.001
Fistula, *n* (%)	16 (19)	10 (44)	5 (16)	1 (3.3) #	<0.001
Valve perforation, *n* (%)	42 (50)	14 (61)	17 (55)	11 (37)	0.173
Pseudoaneurysm, *n* (%)	7 (8.3)	3 (13)	4 (13)	0	0.089
Vegetation Diameter, mm, median (IQR)	13.0 (8)	12.5 (5.9)	11.5 (10.7)	14.5 (6.5)	0.273
LVEF, %, median (IQR)	61 (17)	61 (16)	64 (6)	66 (6) *	0.042
Preoperative laboratory test					
PCT, ng/mL, median (IQR)	0.20 (1.1)	1.10 (2.55)	0.24 (1.34)	0.17 (0.29)	0.128
WBC, 10^9^/L, median (IQR)	8.4 (5.6)	7.1 (2.3)	10.7 (9.7) *	8.7 (4.7)	0.002 &
NEUT, %, median (IQR)	76 (21)	60 (21)	79 (16) *	78 (14) *	<0.001
Monocyte/Lymphocyte, median (IQR)	0.54 (0.69)	0.34 (0.29)	0.98 (1.03) *	0.62 (0.45)	<0.001
PLT, 10^9^/L, median (IQR)	204 (135)	180 (52)	206 (119)	231 (122) *	0.007
Albumin, g/L, median (IQR)	33 (8)	37 (7)	31 (9) *	33 (9)	0.005 &
TBIL, μmol/L, median (IQR)	14 (12)	21 (15)	15 (10)	12 (7)	0.147
DBIL, μmol/L, median (IQR)	4 (5)	5 (6)	4 (3)	3 (5)	0.222
Pro-BNP, median (IQR)	2704 (5230)	3543 (4681)	2019 (8666)	1085 (4423)	0.228
Positive blood culture, *n* (%)	13 (15)	0	7 (23)	6 (20)	0.034
Positive tissue culture, *n*(%)	6 (7)	0	4 (13)	2 (6.7)	0.228

Continuous data are presented as the median (IQR), and categorical data are presented as *n* (%). CBAV Congenital bicuspid aortic valve, LVEF left ventricular ejection fraction, PCT procalcitonin, WBC white blood cell, NUET neutrophil ratio, PLT platelet, TBIL total bilirubin, DBIL direct bilirubin, pro-BNP pro-atrial natriuretic peptide. * Compared with the *C. burnetii* and *Bartonella* endocarditis groups by Dunnett’s test, *p* < 0.05. # Compared with the *C. burnetii* and *Bartonella* endocarditis groups by the chi-square test, *p* < 0.017. & Welch test.

## Data Availability

The microbial genome of *Coxiella burnetii* and *Bartonella* can be downloaded from https://www.ncbi.nlm.nih.gov/sra/PRJNA853129 accessed on 30 June 2022.

## Supplementary Materials

The following supporting information can be downloaded at: https://www.mdpi.com/article/10.3390/jcm11237150/s1, Video S1: Surgical macroscopic view of vegetation and paravalvular abscess in patients with endocarditis caused by *Coxiella burnetii*, *Staphylococcus aureus* and *Streptococcus*; Table S1: Demographic and clinical characteristics of 23 patients; Table S2: Preoperative Transthoracic echocardiography or preoperative transesophageal echography; Table S3:Examniation of the pathogen; Table S4: Metagenomic next-generation (mNGS) summary in the 23 patients.
