# Supplementary material for: Coxiella burnetii and Bartonella Endocarditis Diagnosed by Metagenomic Next-Generation Sequencing
Source: J Clin Med. 2022 Dec 1;11(23):7150. doi: 10.3390/jcm11237150 (PMC9736278; doi:10.3390/jcm11237150)
Supplement: Supplementary file 1 [file jcm-11-07150-s001.zip › Supplementary materials/Supplement 1 Demographic and clinical characteristics of 23 patients.pdf]

Supplement S1

Table S1 Demographic and clinical characteristics of 23 patients

Patient (No.)	Age	Cost (RMB, thousand)	LOS in ICU (D)	LOS in Admission (D)	Coronary Artery Disease	Classification of NYAH	Acute Heart Failure	Arrythmia	Neurological Complication	Surgical Timing (D)	PCT	WBC	NEUT (%)	PLT	ALT	TBIL/DBIL	Serum BUN	SCr	CPB (min)	ACC (min)	Follow-up (months)
1	52	246.25	6	51	Y	IV	Y	N	Stroke	8	-	8.46	46	263	6	5.4/1.5	5.62	715	152	111	37
2	64	172.36	3	22	N	III	N	N	N	12	-	10.69	74	235	29	22.1/7.6	9.68	109	169	126	35
3	59	2615	3	56	Y	III	N	Premature atrial, ventricular contraction	N	12	2	8.73	78	202	25	37/9.6	5.62	55.61	248	195	34
4	44	199.28	4	33	N	III	N	N	Cerebral aneurysm, stroke	13	<0.05	7.10	49	182	22	18.5/3.5	8.33	91.98	171	126	29
5	47	194.96	3	26	N	III	Y	N	Stroke	8	14.00	6.69	65	206	77	22.7/8.5	11.95	94.60	193	155	29
6	36	217.08	5	22	Y	II	N	N	N	< 48h	<0.05	5.01	53	180	18	27.3/6.4	5.99	84.59	113	245	29
7	60	251.19	7	21	N	IV	Y	N	N	3	29.00	5.08	76	34	27	37/10.9	320	66.78	173	115	28
8	56	179.61	4	48	N	III	N	N	N	6	0.07	6.64	51	156	9	12.0/2.5	6.55	79.77	134	101	26
9	59	212.24	2	47	N	III	N	Premature ventricular contraction	N	8	-	8.41	52	330	18	21/5.4	9.71	129.68	181	108	26
10	47	141.54	5	41	N	III	N	N	N	< 24h	0.06	8.06	67	176	24	23.6/4.9	4.93	83.70	112	77	25
11	72	177.75	4	49	Y	II	N	N	N	8	2.61	7.10	39	184	47	16.1/3.1	5.65	95.80	217	148	23
12	67	577.97	22	59	N	III	N	Premature ventricular contraction	N	20	4.27	6.27	59	212	16	11.8/3.1	9.01	122.04	280	179	21
13	64	169.45	3	56	Y	II	N	Atrial fibrillation	N	12	1.43	7.02	60	133	6	15.9/4	5.73	78.97	109	78	21
14	51	172.82	4	9	N	IV	N	N	N	< 48h	1.80	5.13	61	43	27	59.1/25.6	12.21	102.82	170	120	21
15	57	377.54	14	22	N	IV	Y	Atrial fibrillation	N	7	1.85	20.57	87	168	1064	54.7/23.7	14.63	106.56	242	156	21
16	69	737.07	29	38	Y	IV	N	Atrial fibrillation	N	9	-	6.27	55	185	38	14/5.3	8.35	113.66	200	149	20
17	56	492.72	17	20	N	IV	Y	N	N	4	1.10	7.94	82	27	588	245.8/150.5	16.49	226.88	152	111	14
18	55	168.78	2	11	Y	III	N	N	Stroke	7	0.20	7.80	69	196	17	17.2/3	7.74	95.48	169	126	14
19	41	251.31	9	23	N	III	N	N	N	1	7.16	6.01	54	154	32	21.5/8.8	5.41	79.01	246	165	11
20	64	167.81	3	18	N	III	N	N	N	12	0.31	6.08	56	146	16	20.5/4.7	9.92	105.13	100	62	11
21	67	128.05	2	14	N	III	N	N	N	10	<0.05	4.27	60	158	13	7.9/1.5	5.05	113.37	84	113	11
22	56	295.72	7	12	Y	IV	Y	N	N	<48h	0.38	12.14	83	267	12	8.5/3.7	6.16	92.09	178	303	10
23	44	112.375	2	26	Y	III	Y	N	Stroke	21	<0.05	7.13	59	165	34	9.9/2.2	6.58	73.19	124	96	10

LOS Length of stay, ICU Intensive care unit, D Day, NYHA New York Heart Association, PCT Procalcitonin, WBC White blood cell, NUET Neutrophile ratio, PLT Platelet, ALT Alanine aminotransferase, TBIL Total bilirubin, BUN Blood urea nitrogen, SCr Serum creatinine, Y Yes, N No, “-” No done, CPB Cardiac pulmonary bypass, ACC Aorta cross-clamp.

Table S2 Preoperative Transthoracic Echocardiography or Preoperative Transesophageal Echocardiography

Patient	CBAV	AI	AS	MI	MS	TI	PASP (mmHg)	EF (%)	Vegetation Diameter (mm)	Abscess	Abscess Diameter (mm)	Valvular Perforation	Fistula	Pseudoaneurysm
1	Y	Seve	Mild	Mild			-	57	14.8	Y	12*8.3, 25*14	AV	N	N
2	N	Seve	Mod	Mod	Mod	Mod	43	64	14	N			N	N
3	Y	Seve	Mild	Mild		Mod	74	62	8.8	Y	47*20	AV	Y	N
4	N	Seve		Seve	Mild	Seve	67	66	9	N		AV	N	N
5	Y	Seve	Seve	Mild		Mild	61	62	9	Y	27*19<br>20*15	AV	Y	Y
6	Y	Seve	Mild	Mod		Mod	67	45	17	Y	5*3		Y	N
7	Y	Seve	Mod	Mod		Mod	71	68	19	Y	28*13, 18*14	AV	N	N
8		Seve	Seve	Seve	Seve	Mild	65	65	13	Y	12*10	AV	N	Y
9	Y	Seve	Mod	Mod		Mod	57	43	10	Y	21*12<br>13*10	AV	Y	N
10	Y	Seve		Mod		Mild	52	60	6	Y	15*13	AV	N	N
11	Y	Seve	Seve					91	Obscure	N			N	N
12	N	Seve	Mod	Seve		Seve	50	31	Obscure	N			N	N
13	Y	Mild	Mod	Mild		Mild	31	53	6	N			N	N
14	N	Seve		Seve		Seve	83	49	12	Y	26*13, 16*6	AV	Y	N
15	Y	Mod	Seve	Mild		Mild	50	20	13	Y	40	AV	Y	N
16	N	Mod	Seve	Mild	Mod	Mild	25	61	14.8	Y	12*8.3, 25*14	AV	Y	N
17	Y	Seve	Seve	Seve		Seve	99	51	17	Y	21*21, 12*5		Y	N
18	Y	Seve	Mid	Seve		Seve	77	66	19	Y	30*23	AV	Y	N
19	Y	Seve	Mod	Mild		Mild	54	45	Obscure	Y	32*24		Y	N
20	Y	Seve	Mod	Mod				65	0.5	N			N	Y
21	N			Seve		Mod	47	61	12	N			N	N
22	N	Seve		Seve		Seve	50	68	12, 13, 13	N		AV	N	N
23	Y	Seve	Seve	Mild		Mod	57	87	Obscure	N		AV	N	N

CBAV Congenital bicuspid aortic valve, AI Aortic valve insufficiency, AS Aortic valve stenosis, MI Mitral valve insufficiency, MS Mitral valve stenosis, TI Tricuspid valve insufficiency, PASP Pulmonary arterial systolic pressure, Y Yes, N No, Seve Severe, Mod Moderate, Mil Mild, AV Aortic valve.

Table S3 Examination of the Pathogen

Patient No.	BC	RTI (D)	VC	RTI (D)	mNGS of Venous Blood	*Unique Pathogen Read-pairs, No. (%Suspected Pathogen Read-pairs)	mNGS of Arterial Blood	*Unique Pathogen Read-pairs, No. (%Suspected Pathogen Read-pairs)	mNGS of Tissue	*Unique Pathogen Read-pairs, No. (%Suspected Pathogen Read-pairs)	RTI (D)
1	N	6	N	6	N		N		*C. burnetii*	40495(62.24)	1
2	-		-		*C. burnetii*	7(0.05)	*C. burnetii*	17(0.08)	-		1
3	N	6	N	6	N		N		*C. burnetii*	1091631(97.83)	1
4	N	6	N	6	-		-		*B. quintana*	314369(74.94)	1
5	N	6	N	6	N		*C. burnetii*	6(0.03)	*C. burnetii*	84059(59.76)	1
6	N	6	N	6	*C. burnetii*	4 (0.01)	*C. burnetii*	3(0.01)	*C. burnetii*	32078(61.79)	1
7	N	6	N	6	*C. burnetii*	12(0.05)	*C. burnetii*	3(0.02)	*C. burnetii*	112026(81.83)	1
8	N	6	N	6	*C. burnetii*	63(0.35)	*C. burnetii*	48(0.24)	*C. burnetii*	46669(84.24)	1
9	N	6	N	6	*C. burnetii*	3(0.01)	*C. burnetii*	3(0.01)	*C. burnetii*	21008(45.92)	1
10	N	6	N	6	*C. burnetii*	4(0.04)	*C. burnetii*	4(0.03)	*C. burnetii*	131(1.74)	1
11	-		-		*C. burnetii*	7(0.01)	*C. burnetii*	4(0.01)	*C. burnetii*	195(0.46)	1
12	N	6	N	6	*C. burnetii*	7(0.02)	N		N		1
13	N	6	N	6	N		N		*C. burneti*	242(1.91)	1
14	N	6	N	6	*C. burnetii*	31(0.09)	*C. burnetii*	32(0.11)	*C. burnetii*	202036(86.74)	1
15	N	6	N	5	*C. burnetii*	10(0.03)	*C. burnetii*	11(0.03)	*C. burnetii*	543(4.87)	2
16	-	-	N	6	*C. burnetii*	31(0.05)	*C. burnetii*	18(0.03)	*C. burnetii*	560(0.84)	1
17	N	6	N	6	*C. burnetii*	68(0.08)	*C. burnetii*	73(0.10)	*C. burnetii*	1974(3.63)	1
							Streptococcus gordonii	3(< 0.01)	*Streptococc us gordonii*	45(0.08)	
18	N	6	N	6	*C. burnetii*	28(0.038)	*C. burnetii*	17 (0.02)	*C. burnetii*	11378(18.56)	1
19	N	6	N	6	*C. burnetii*	909(1.45)	*C. burnetii*	971(1.01)	*C. burnetii*	5046(22.09)	1
20	N	6	N	6	*C. burnetii*	4(2.34)	*C. burnetii*	3(0.13)	*C. burnetii*	4086675(99.13)	1
21	N	6	N	6	*C. burnetii*	119(1.52)	*C. burnetii*	17(1.21)	*C. burnetii*	204116(98.97)	1
22	N	6	N	6	*B. henselae*	1134(28.34)	*B. henselae*	656(21.93)	*B. henselae*	2630405(62.64)	1
23	N	6	N	6	*C. burnetii*	24(2.22)	*C. burnetii*	25(1.72)	*C. burnetii*	1512(40.40)	1

RT Reporting time interval, D Day, mNGS Metagenomic next-generation sequence, N Negative, "-" No done, *C. burnetii Coxiella burnetii, B. quintana Bartonella quintana*. * Metagenomic Next-generation Sequencing (mNGS) Summary in the 22 patients was described in the **Table 4.**

Table S4 Metagenomic Next-generation Sequencing (mNGS) Summary in the 23 patients

Patient No.	mNGS of Venous Blood			mNGS of Arterial Blood			mNGS of Excised Valve Tissue		
	Unique Human Nonredundant Read-Pairs, No.	Unique Nonhuman Nonredundant Read-Pairs, No.	Unique suspected Pathogen Read-pairs, No. (% Nonhuman Read-Pairs)	Unique Human Nonredundant Read-Pairs, No.	Unique Nonhuman Nonredundant Read-Pairs, No.	Unique suspected Pathogen Read-pairs, No. (% Nonhuman Read-Pairs)	Unique Human Nonredundant Read-Pairs, No.	Unique Nonhuman Nonredundant Read-Pairs, No.	Unique suspected Pathogen Read-pairs, No. (% Nonhuman Read-Pairs)
1	14495520	413476	13059(3.16)	14431879	412114	12249(2.97)	10292802	165853	65067(39.23)
2	7691690	491789	15557(3.16)	10162576	637825	20358(3.19)	-	-	-
3	8700381	1695227	50139(2.96)	8195825	1337497	40467(3.03)	12372422	1646087	1115874(67.79)
4	-	-	-	-	-	-	14750280	956928	419514(43.84)
5	13590085	384795	18429(4.79)	16867380	401876	21032(5.23)	16767900	1171801	140659(12.00)
6	9538066	1108004	33758(3.05)	9855286	794095	22830(2.87)	14206757	354587	51914(14.64)
7	12467203	492020	24617(5.00)	9644690	617555	19986(3.24)	18957628	513095	137788(26.85)
8	10072757	459834	18088(3.93)	7857014	526026	20104(3.82)	10737663	227092	55403(24.40)
9	9633431	760221	27166(3.57)	10448081	1021361	37530(3.67)	13972580	466314	45750(9.81)
10	8076569	267592	10947(4.09)	7296733	278689	11983(4.30)	11167057	146693	7527(5.13)
11	20731250	1475921	75375(5.11)	16273331	1445594	69159(4.78)	30100695	674768	42635(6.32)
12	19637097	640422	36351(5.68)	18754358	583612	34121(5.58)	18040152	157306	9453(6.01)
13	21063360	981289	45850(4.67)	24713478	1061504	50180(4.73)	29012479	206495	12644(6.12)
14	19610696	1198911	36156(3.02)	16643788	909125	28868(3.18)	19146985	631510	232930(36.88)
15	17897162	482786	29506(6.11)	17608489	767830	35993(4.69)	19400743	162069	11152(6.88)
16	31648270	1139320	65760(5.77)	30330328	951448	55371(5.82)	29908284	1037182	66654(6.43)
17	41571061	1377365	90168(6.55)	41089335	1165517	72480(6.22)	39566384	819235	54350(6.63)
18	23804639	1308862	74462(5.69)	21652773	1471439	74784(5.08)	25515260	969681	61315(6.32)
19	22379873	962019	62815(6.53)	25519498	1514405	96613(6.38)	27097626	236938	22846(9.64)
20	93645	2419	171(7.07)	3162869	26101	2231(8.55)	12445221	5128477	4122668(80.39)
21	16768004	429184	1253(0.29)	17764400	523808	1405(0.27)	26002502	446102	206235(46.23)
22	22637965	664292	4002(0.60)	18152133	599574	2992(0.50)	14485835	5552461	4198919(75.62)
23	9526907	361473	1081(0.30)	11067320	508288	1454(0.29)	17277467	368911	3743(1.01)
